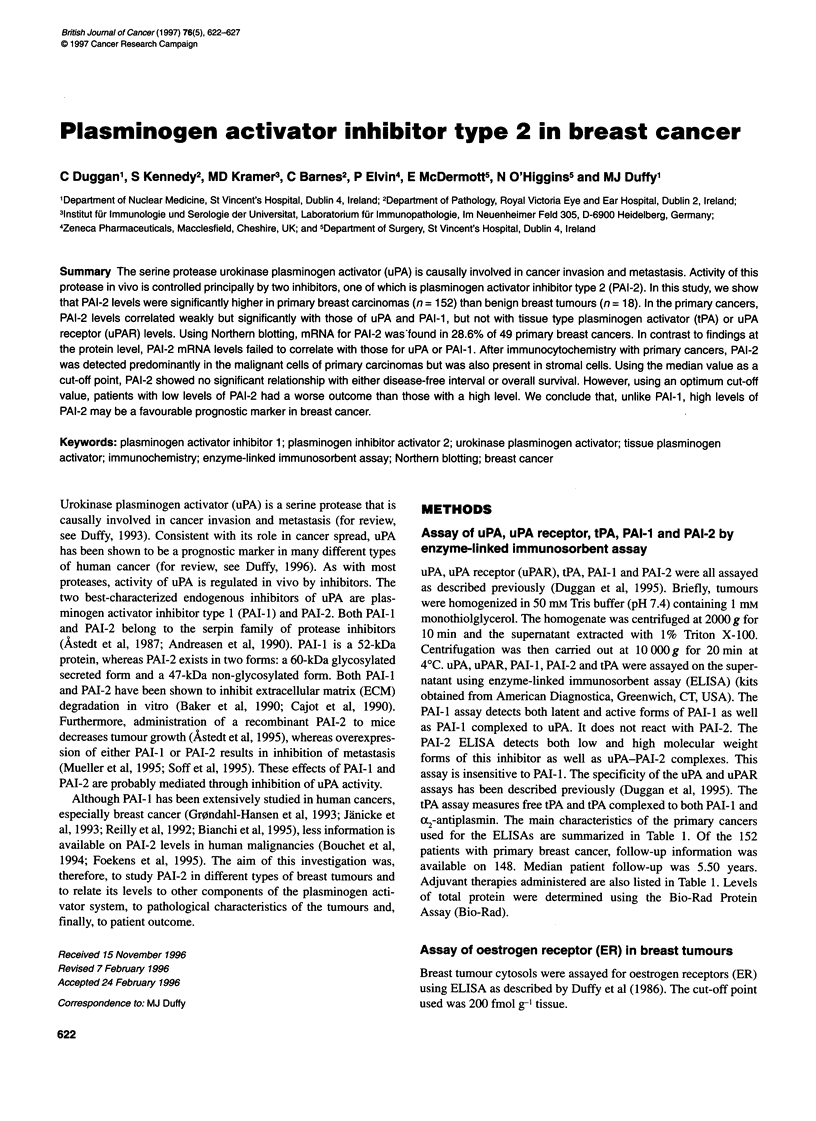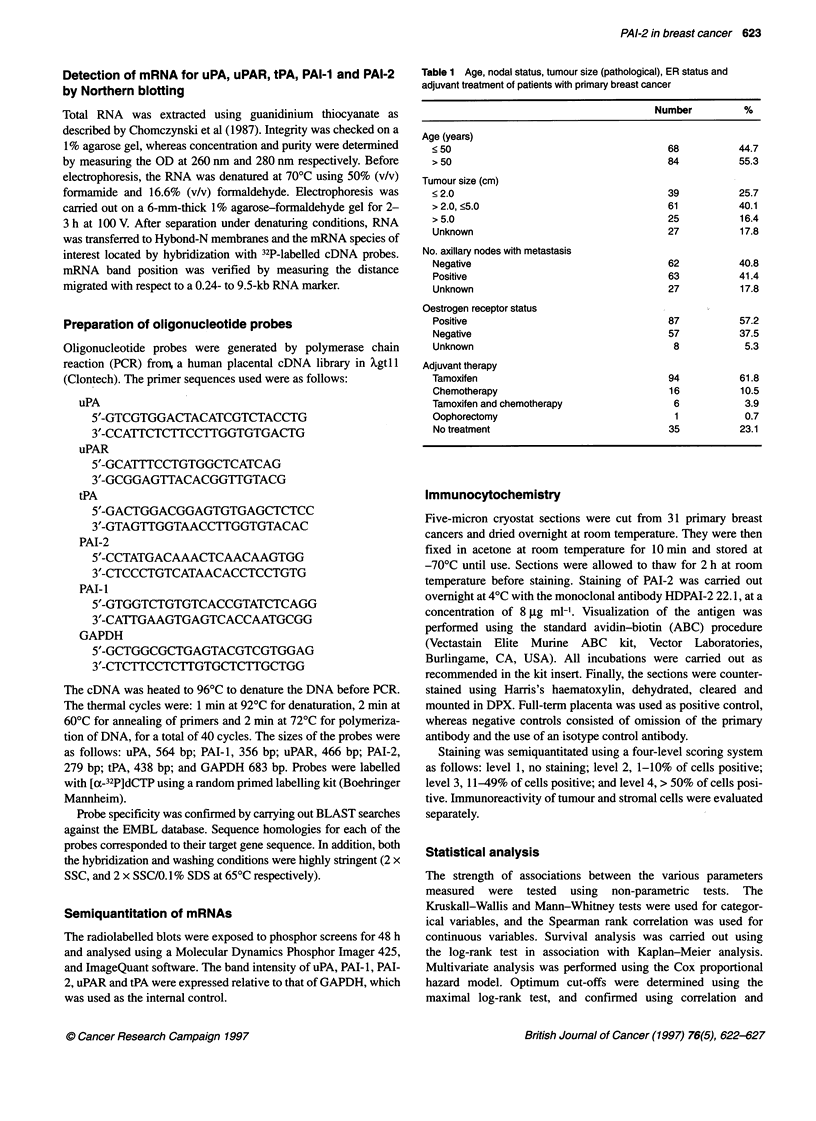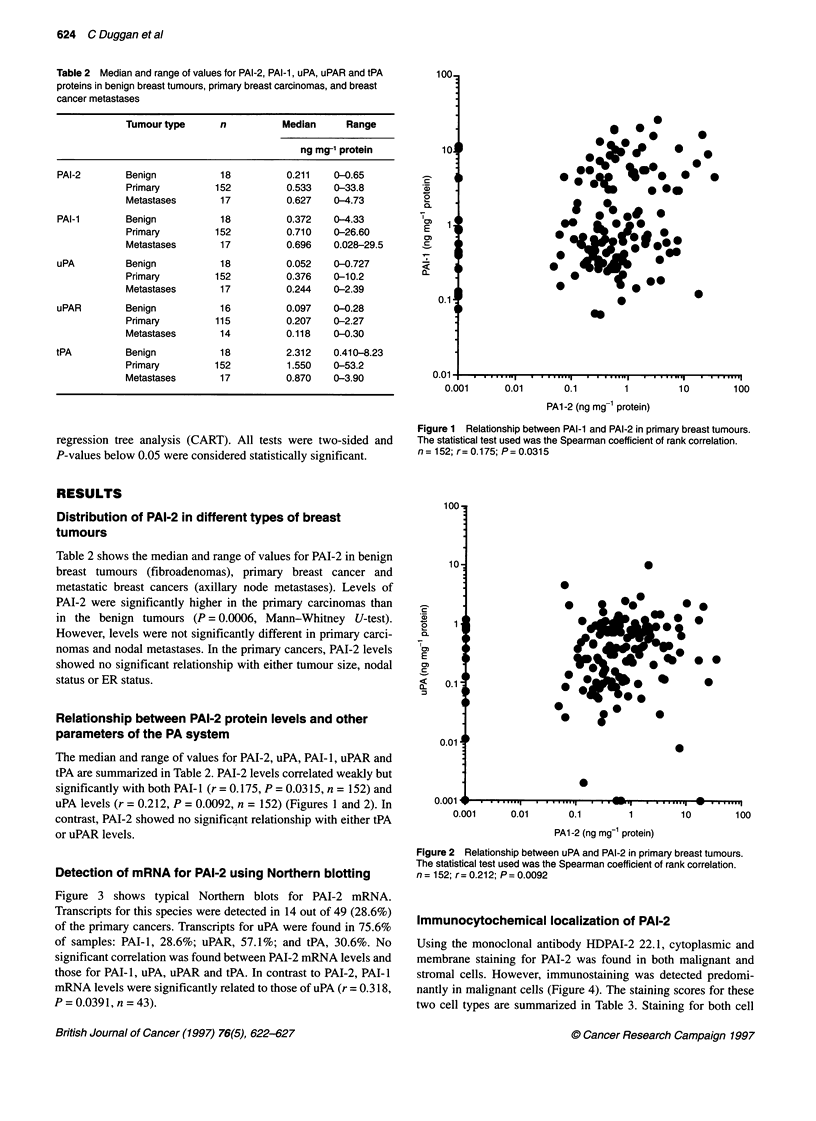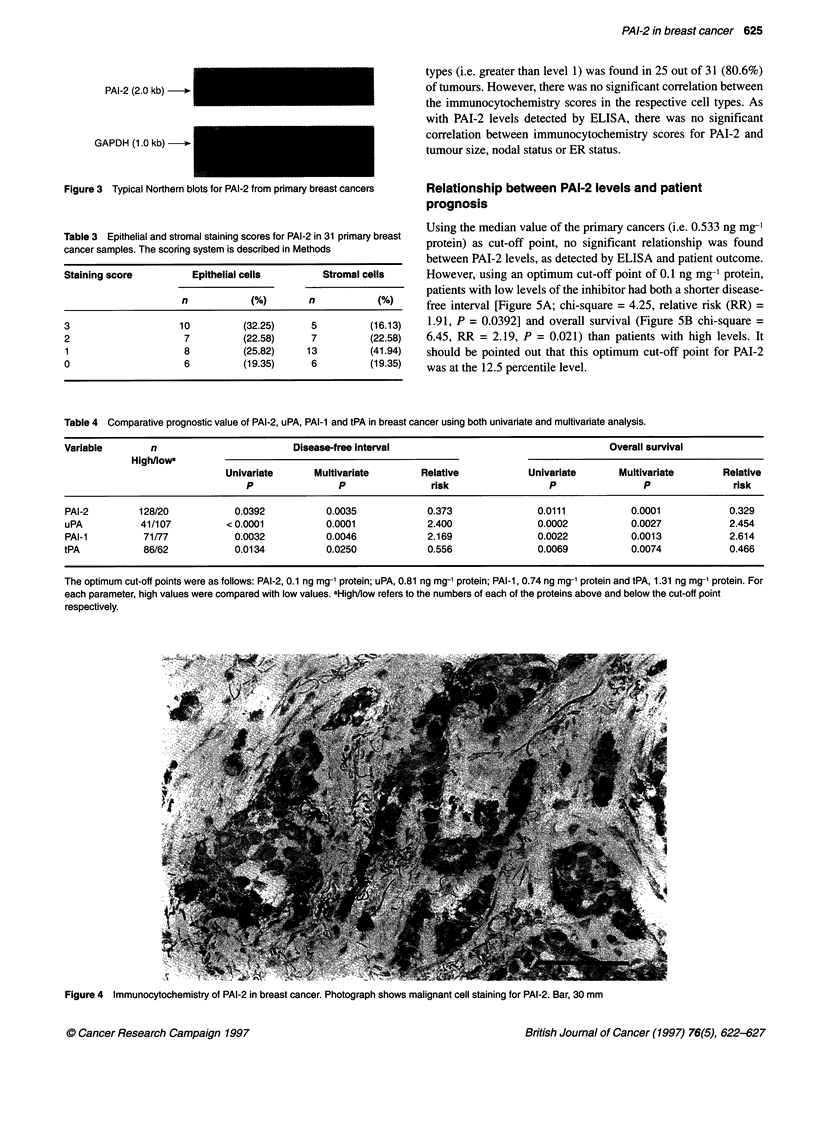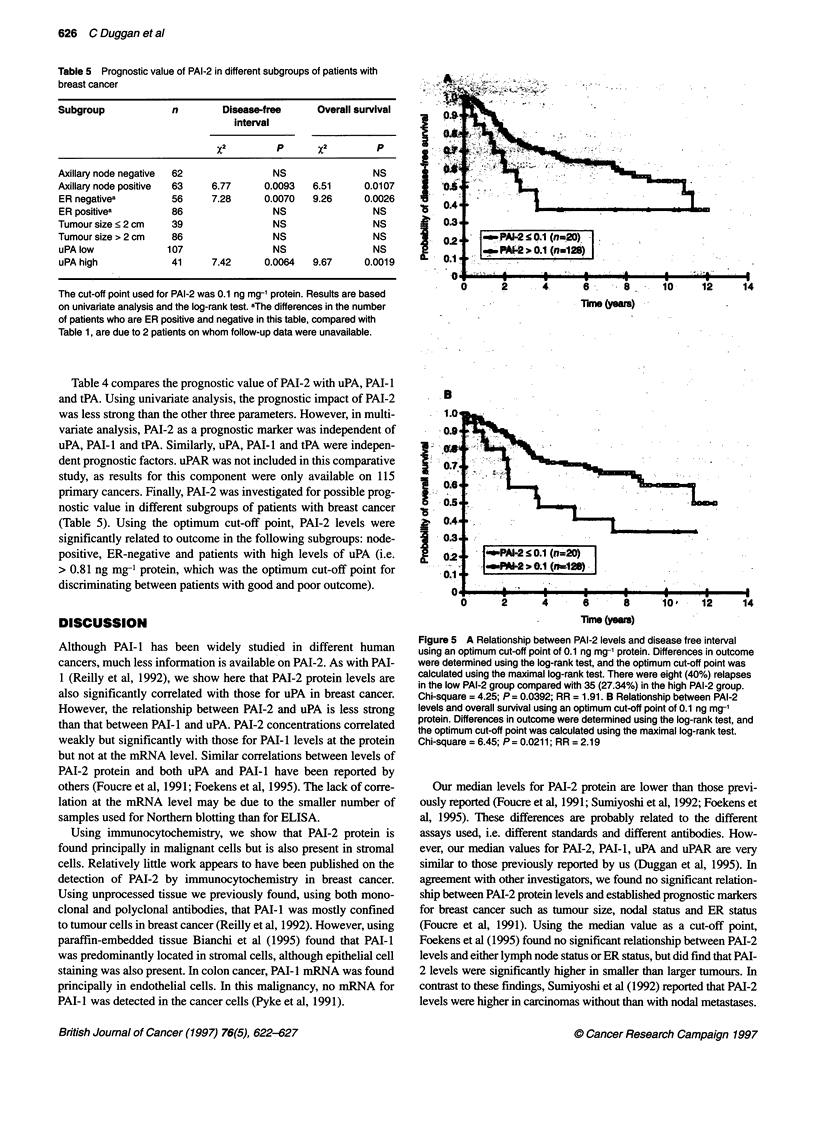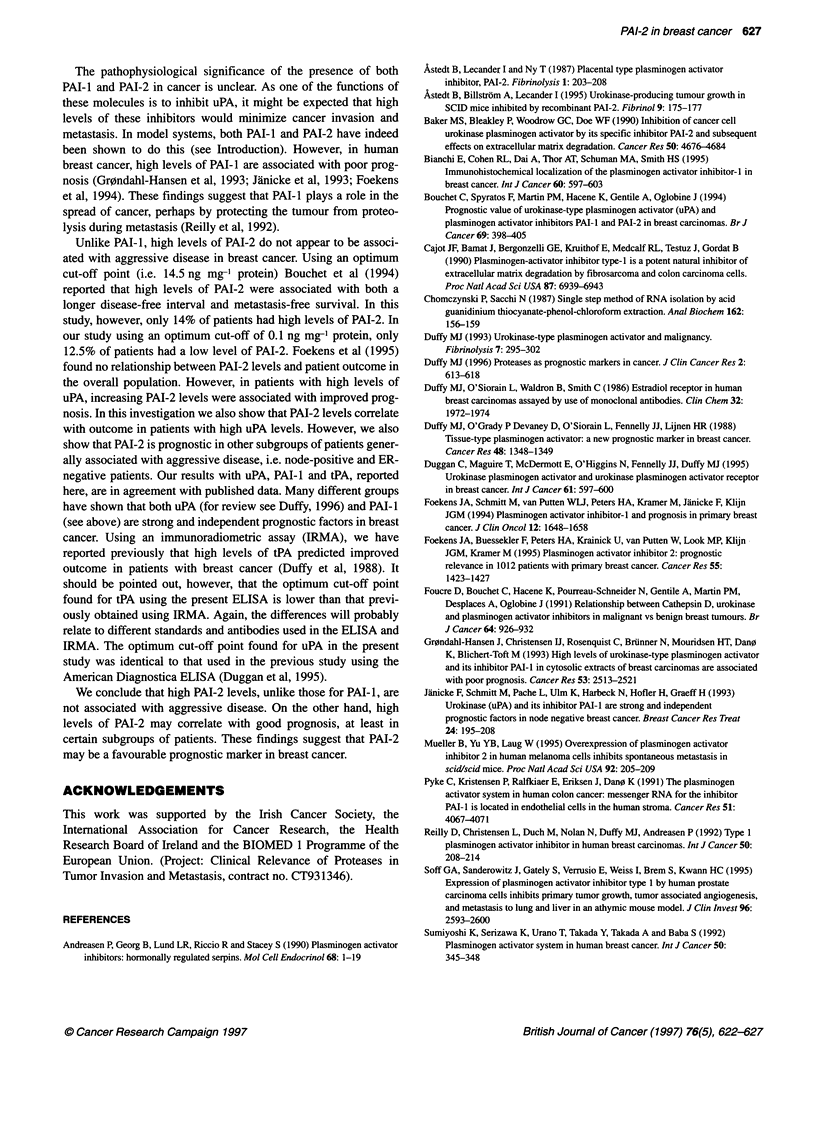# Plasminogen activator inhibitor type 2 in breast cancer.

**DOI:** 10.1038/bjc.1997.435

**Published:** 1997

**Authors:** C. Duggan, S. Kennedy, M. D. Kramer, C. Barnes, P. Elvin, E. McDermott, N. O'Higgins, M. J. Duffy

**Affiliations:** Department of Nuclear Medicine, St Vincent's Hospital, Dublin, Ireland.

## Abstract

**Images:**


British Journal of Cancer (1997) 76(5), 622–627
© 1997 Cancer Research Campaign

### Plasminogen activator inhibitor type 2 in breast cancer

**C Duggan[1], S Kennedy[2], MD Kramer[3], C Barnes[2], P Elvin[4], E McDermott[5], N O'Higgins[5] and MJ Duffy[1]**

[1]Department of Nuclear Medicine, St Vincent's Hospital, Dublin 4, Ireland; [2]Department of Pathology, Royal Victoria Eye and Ear Hospital, Dublin 2, Ireland; [3]Institut für Immunologie und Serologie der Universitat, Laboratorium für Immunopathologie, Im Neuenheimer Feld 305, D-6900 Heidelberg, Germany; [4]Zeneca Pharmaceuticals, Macclesfield, Cheshire, UK; and [5]Department of Surgery, St Vincent's Hospital, Dublin 4, Ireland

**Summary** The serine protease urokinase plasminogen activator (uPA) is causally involved in cancer invasion and metastasis. Activity of this protease in vivo is controlled principally by two inhibitors, one of which is plasminogen activator inhibitor type 2 (PAI-2). In this study, we show that PAI-2 levels were significantly higher in primary breast carcinomas ($n = 152$) than benign breast tumours ($n = 18$). In the primary cancers, PAI-2 levels correlated weakly but significantly with those of uPA and PAI-1, but not with tissue type plasminogen activator (tPA) or uPA receptor (uPAR) levels. Using Northern blotting, mRNA for PAI-2 was found in 28.6% of 49 primary breast cancers. In contrast to findings at the protein level, PAI-2 mRNA levels failed to correlate with those for uPA or PAI-1. After immunocytochemistry with primary cancers, PAI-2 was detected predominantly in the malignant cells of primary carcinomas but was also present in stromal cells. Using the median value as a cut-off point, PAI-2 showed no significant relationship with either disease-free interval or overall survival. However, using an optimum cut-off value, patients with low levels of PAI-2 had a worse outcome than those with a high level. We conclude that, unlike PAI-1, high levels of PAI-2 may be a favourable prognostic marker in breast cancer.

**Keywords:** plasminogen activator inhibitor 1; plasminogen inhibitor activator 2; urokinase plasminogen activator; tissue plasminogen activator; immunochemistry; enzyme-linked immunosorbent assay; Northern blotting; breast cancer

Urokinase plasminogen activator (uPA) is a serine protease that is causally involved in cancer invasion and metastasis (for review, see Duffy, 1993). Consistent with its role in cancer spread, uPA has been shown to be a prognostic marker in many different types of human cancer (for review, see Duffy, 1996). As with most proteases, activity of uPA is regulated in vivo by inhibitors. The two best-characterized endogenous inhibitors of uPA are plasminogen activator inhibitor type 1 (PAI-1) and PAI-2. Both PAI-1 and PAI-2 belong to the serpin family of protease inhibitors (Åstedt et al, 1987; Andreasen et al, 1990). PAI-1 is a 52-kDa protein, whereas PAI-2 exists in two forms: a 60-kDa glycosylated secreted form and a 47-kDa non-glycosylated form. Both PAI-1 and PAI-2 have been shown to inhibit extracellular matrix (ECM) degradation in vitro (Baker et al, 1990; Cajot et al, 1990). Furthermore, administration of a recombinant PAI-2 to mice decreases tumour growth (Åstedt et al, 1995), whereas overexpression of either PAI-1 or PAI-2 results in inhibition of metastasis (Mueller et al, 1995; Soff et al, 1995). These effects of PAI-1 and PAI-2 are probably mediated through inhibition of uPA activity.

Although PAI-1 has been extensively studied in human cancers, especially breast cancer (Grøndahl-Hansen et al, 1993; Jänicke et al, 1993; Reilly et al, 1992; Bianchi et al, 1995), less information is available on PAI-2 levels in human malignancies (Bouchet et al, 1994; Foekens et al, 1995). The aim of this investigation was, therefore, to study PAI-2 in different types of breast tumours and to relate its levels to other components of the plasminogen activator system, to pathological characteristics of the tumours and, finally, to patient outcome.

*Received 15 November 1996*
*Revised 7 February 1996*
*Accepted 24 February 1996*

*Correspondence to:* MJ Duffy

### METHODS

### Assay of uPA, uPA receptor, tPA, PAI-1 and PAI-2 by enzyme-linked immunosorbent assay uPA, uPA receptor (uPAR), tPA, PAI-1 and PAI-2 were all assayed as described previously (Duggan et al, 1995). Briefly, tumours were homogenized in 50 mM Tris buffer (pH 7.4) containing 1 mM monothiolglycerol. The homogenate was centrifuged at 2000 $g$ for 10 min and the supernatant extracted with 1% Triton X-100. Centrifugation was then carried out at 10 000 $g$ for 20 min at 4°C. uPA, uPAR, PAI-1, PAI-2 and tPA were assayed on the supernatant using enzyme-linked immunosorbent assay (ELISA) (kits obtained from American Diagnostica, Greenwich, CT, USA). The PAI-1 assay detects both latent and active forms of PAI-1 as well as PAI-1 complexed to uPA. It does not react with PAI-2. The PAI-2 ELISA detects both low and high molecular weight forms of this inhibitor as well as uPA–PAI-2 complexes. This assay is insensitive to PAI-1. The specificity of the uPA and uPAR assays has been described previously (Duggan et al, 1995). The tPA assay measures free tPA and tPA complexed to both PAI-1 and $\alpha_2$-antiplasmin. The main characteristics of the primary cancers used for the ELISAs are summarized in Table 1. Of the 152 patients with primary breast cancer, follow-up information was available on 148. Median patient follow-up was 5.50 years. Adjuvant therapies administered are also listed in Table 1. Levels of total protein were determined using the Bio-Rad Protein Assay (Bio-Rad).

### Assay of oestrogen receptor (ER) in breast tumours

Breast tumour cytosols were assayed for oestrogen receptors (ER) using ELISA as described by Duffy et al (1986). The cut-off point used was 200 fmol $g^{-1}$ tissue.

### Detection of mRNA for uPA, uPAR, tPA, PAI-1 and PAI-2 by Northern blotting

Total RNA was extracted using guanidinium thiocyanate as described by Chomczynski et al (1987). Integrity was checked on a 1% agarose gel, whereas concentration and purity were determined by measuring the OD at 260 nm and 280 nm respectively. Before electrophoresis, the RNA was denatured at 70°C using 50% (v/v) formamide and 16.6% (v/v) formaldehyde. Electrophoresis was carried out on a 6-mm-thick 1% agarose–formaldehyde gel for 2–3 h at 100 V. After separation under denaturing conditions, RNA was transferred to Hybond-N membranes and the mRNA species of interest located by hybridization with $^{32}P$-labelled cDNA probes. mRNA band position was verified by measuring the distance migrated with respect to a 0.24- to 9.5-kb RNA marker.

### Preparation of oligonucleotide probes

Oligonucleotide probes were generated by polymerase chain reaction (PCR) from a human placental cDNA library in λgt11 (Clontech). The primer sequences used were as follows:

uPA
5′-GTCGTGGACTACATCGTCTACCTG
3′-CCATTCTCTTCCTTGGTGTGACTG
uPAR
5′-GCATTTCCTGTGGCTCATCAG
3′-GCGGAGTTACACGGTTGTACG
tPA
5′-GACTGGACGGAGTGTGAGCTCTCC
3′-GTAGTTGGTAACCTTGGTGTACAC
PAI-2
5′-CCTATGACAAACTCAACAAGTGG
3′-CTCCCTGTCATAACACCTCCTGTG
PAI-1
5′-GTGGTCTGTGTCACCGTATCTCAGG
3′-CATTGAAGTGAGTCACCAATGCGG
GAPDH
5′-GCTGGCGCTGAGTACGTCGTGGAG
3′-CTCTTCCTCTTGTGCTCTTGCTGG

The cDNA was heated to 96°C to denature the DNA before PCR. The thermal cycles were: 1 min at 92°C for denaturation, 2 min at 60°C for annealing of primers and 2 min at 72°C for polymerization of DNA, for a total of 40 cycles. The sizes of the probes were as follows: uPA, 564 bp; PAI-1, 356 bp; uPAR, 466 bp; PAI-2, 279 bp; tPA, 438 bp; and GAPDH 683 bp. Probes were labelled with [α-$^{32}P$]dCTP using a random primed labelling kit (Boehringer Mannheim).

Probe specificity was confirmed by carrying out BLAST searches against the EMBL database. Sequence homologies for each of the probes corresponded to their target gene sequence. In addition, both the hybridization and washing conditions were highly stringent (2 × SSC, and 2 × SSC/0.1% SDS at 65°C respectively).

### Semiquantitation of mRNAs

The radiolabelled blots were exposed to phosphor screens for 48 h and analysed using a Molecular Dynamics Phosphor Imager 425, and ImageQuant software. The band intensity of uPA, PAI-1, PAI-2, uPAR and tPA were expressed relative to that of GAPDH, which was used as the internal control.

**Table 1** Age, nodal status, tumour size (pathological), ER status and adjuvant treatment of patients with primary breast cancer

| | Number | % |
|---|---|---|
| Age (years) | | |
| ≤ 50 | 68 | 44.7 |
| > 50 | 84 | 55.3 |
| Tumour size (cm) | | |
| ≤ 2.0 | 39 | 25.7 |
| > 2.0, ≤5.0 | 61 | 40.1 |
| > 5.0 | 25 | 16.4 |
| Unknown | 27 | 17.8 |
| No. axillary nodes with metastasis | | |
| Negative | 62 | 40.8 |
| Positive | 63 | 41.4 |
| Unknown | 27 | 17.8 |
| Oestrogen receptor status | | |
| Positive | 87 | 57.2 |
| Negative | 57 | 37.5 |
| Unknown | 8 | 5.3 |
| Adjuvant therapy | | |
| Tamoxifen | 94 | 61.8 |
| Chemotherapy | 16 | 10.5 |
| Tamoxifen and chemotherapy | 6 | 3.9 |
| Oophorectomy | 1 | 0.7 |
| No treatment | 35 | 23.1 |

### Immunocytochemistry

Five-micron cryostat sections were cut from 31 primary breast cancers and dried overnight at room temperature. They were then fixed in acetone at room temperature for 10 min and stored at –70°C until use. Sections were allowed to thaw for 2 h at room temperature before staining. Staining of PAI-2 was carried out overnight at 4°C with the monoclonal antibody HDPAI-2 22.1, at a concentration of 8 μg $ml^{-1}$. Visualization of the antigen was performed using the standard avidin–biotin (ABC) procedure (Vectastain Elite Murine ABC kit, Vector Laboratories, Burlingame, CA, USA). All incubations were carried out as recommended in the kit insert. Finally, the sections were counter-stained using Harris's haematoxylin, dehydrated, cleared and mounted in DPX. Full-term placenta was used as positive control, whereas negative controls consisted of omission of the primary antibody and the use of an isotype control antibody.

Staining was semiquantitated using a four-level scoring system as follows: level 1, no staining; level 2, 1–10% of cells positive; level 3, 11–49% of cells positive; and level 4, > 50% of cells positive. Immunoreactivity of tumour and stromal cells were evaluated separately.

### Statistical analysis

The strength of associations between the various parameters measured were tested using non-parametric tests. The Kruskall–Wallis and Mann–Whitney tests were used for categorical variables, and the Spearman rank correlation was used for continuous variables. Survival analysis was carried out using the log-rank test in association with Kaplan–Meier analysis. Multivariate analysis was performed using the Cox proportional hazard model. Optimum cut-offs were determined using the maximal log-rank test, and confirmed using correlation and

© Cancer Research Campaign 1997

Table 2 Median and range of values for PAI-2, PAI-1, uPA, uPAR and tPA proteins in benign breast tumours, primary breast carcinomas, and breast cancer metastases

| | Tumour type | n | Median | Range |
|---|---|---|---|---|
| | | | ng mg$^{-1}$ protein | |
| PAI-2 | Benign | 18 | 0.211 | 0–0.65 |
| | Primary | 152 | 0.533 | 0–33.8 |
| | Metastases | 17 | 0.627 | 0–4.73 |
| PAI-1 | Benign | 18 | 0.372 | 0–4.33 |
| | Primary | 152 | 0.710 | 0–26.60 |
| | Metastases | 17 | 0.696 | 0.028–29.5 |
| uPA | Benign | 18 | 0.052 | 0–0.727 |
| | Primary | 152 | 0.376 | 0–10.2 |
| | Metastases | 17 | 0.244 | 0–2.39 |
| uPAR | Benign | 16 | 0.097 | 0–0.28 |
| | Primary | 115 | 0.207 | 0–2.27 |
| | Metastases | 14 | 0.118 | 0–0.30 |
| tPA | Benign | 18 | 2.312 | 0.410–8.23 |
| | Primary | 152 | 1.550 | 0–53.2 |
| | Metastases | 17 | 0.870 | 0–3.90 |

regression tree analysis (CART). All tests were two-sided and P-values below 0.05 were considered statistically significant.

### RESULTS

### Distribution of PAI-2 in different types of breast tumours

Table 2 shows the median and range of values for PAI-2 in benign breast tumours (fibroadenomas), primary breast cancer and metastatic breast cancers (axillary node metastases). Levels of PAI-2 were significantly higher in the primary carcinomas than in the benign tumours ($P = 0.0006$, Mann–Whitney U-test). However, levels were not significantly different in primary carcinomas and nodal metastases. In the primary cancers, PAI-2 levels showed no significant relationship with either tumour size, nodal status or ER status.

### Relationship between PAI-2 protein levels and other parameters of the PA system

The median and range of values for PAI-2, uPA, PAI-1, uPAR and tPA are summarized in Table 2. PAI-2 levels correlated weakly but significantly with both PAI-1 ($r = 0.175$, $P = 0.0315$, $n = 152$) and uPA levels ($r = 0.212$, $P = 0.0092$, $n = 152$) (Figures 1 and 2). In contrast, PAI-2 showed no significant relationship with either tPA or uPAR levels.

### Detection of mRNA for PAI-2 using Northern blotting

Figure 3 shows typical Northern blots for PAI-2 mRNA. Transcripts for this species were detected in 14 out of 49 (28.6%) of the primary cancers. Transcripts for uPA were found in 75.6% of samples: PAI-1, 28.6%; uPAR, 57.1%; and tPA, 30.6%. No significant correlation was found between PAI-2 mRNA levels and those for PAI-1, uPA, uPAR and tPA. In contrast to PAI-2, PAI-1 mRNA levels were significantly related to those of uPA ($r = 0.318$, $P = 0.0391$, $n = 43$).

Figure 1 Relationship between PAI-1 and PAI-2 in primary breast tumours. The statistical test used was the Spearman coefficient of rank correlation. $n = 152$; $r = 0.175$; $P = 0.0315$

Figure 2 Relationship between uPA and PAI-2 in primary breast tumours. The statistical test used was the Spearman coefficient of rank correlation. $n = 152$; $r = 0.212$; $P = 0.0092$

### Immunocytochemical localization of PAI-2

Using the monoclonal antibody HDPAI-2 22.1, cytoplasmic and membrane staining for PAI-2 was found in both malignant and stromal cells. However, immunostaining was detected predominantly in malignant cells (Figure 4). The staining scores for these two cell types are summarized in Table 3. Staining for both cell

© Cancer Research Campaign 1997

**Figure 3** Typical Northern blots for PAI-2 from primary breast cancers

**Table 3** Epithelial and stromal staining scores for PAI-2 in 31 primary breast cancer samples. The scoring system is described in Methods

| Staining score | Epithelial cells | | Stromal cells | |
|---|---|---|---|---|
| | *n* | (%) | *n* | (%) |
| 3 | 10 | (32.25) | 5 | (16.13) |
| 2 | 7 | (22.58) | 7 | (22.58) |
| 1 | 8 | (25.82) | 13 | (41.94) |
| 0 | 6 | (19.35) | 6 | (19.35) |

types (i.e. greater than level 1) was found in 25 out of 31 (80.6%) of tumours. However, there was no significant correlation between the immunocytochemistry scores in the respective cell types. As with PAI-2 levels detected by ELISA, there was no significant correlation between immunocytochemistry scores for PAI-2 and tumour size, nodal status or ER status.

### Relationship between PAI-2 levels and patient prognosis

Using the median value of the primary cancers (i.e. 0.533 ng $mg^{-1}$ protein) as cut-off point, no significant relationship was found between PAI-2 levels, as detected by ELISA and patient outcome. However, using an optimum cut-off point of 0.1 ng $mg^{-1}$ protein, patients with low levels of the inhibitor had both a shorter disease-free interval [Figure 5A; chi-square = 4.25, relative risk (RR) = 1.91, $P = 0.0392$] and overall survival (Figure 5B chi-square = 6.45, RR = 2.19, $P = 0.021$) than patients with high levels. It should be pointed out that this optimum cut-off point for PAI-2 was at the 12.5 percentile level.

**Table 4** Comparative prognostic value of PAI-2, uPA, PAI-1 and tPA in breast cancer using both univariate and multivariate analysis.

| Variable | *n* High/low[a] | Disease-free interval | | | Overall survival | | |
|---|---|---|---|---|---|---|---|
| | | Univariate *P* | Multivariate *P* | Relative risk | Univariate *P* | Multivariate *P* | Relative risk |
| PAI-2 | 128/20 | 0.0392 | 0.0035 | 0.373 | 0.0111 | 0.0001 | 0.329 |
| uPA | 41/107 | < 0.0001 | 0.0001 | 2.400 | 0.0002 | 0.0027 | 2.454 |
| PAI-1 | 71/77 | 0.0032 | 0.0046 | 2.169 | 0.0022 | 0.0013 | 2.614 |
| tPA | 86/62 | 0.0134 | 0.0250 | 0.556 | 0.0069 | 0.0074 | 0.466 |

The optimum cut-off points were as follows: PAI-2, 0.1 ng $mg^{-1}$ protein; uPA, 0.81 ng $mg^{-1}$ protein; PAI-1, 0.74 ng $mg^{-1}$ protein and tPA, 1.31 ng $mg^{-1}$ protein. For each parameter, high values were compared with low values. [a]High/low refers to the numbers of each of the proteins above and below the cut-off point respectively.

**Figure 4** Immunocytochemistry of PAI-2 in breast cancer. Photograph shows malignant cell staining for PAI-2. Bar, 30 mm

**Table 5** Prognostic value of PAI-2 in different subgroups of patients with breast cancer

| Subgroup | n | Disease-free interval | | Overall survival | |
|---|---|---|---|---|---|
| | | $\chi^2$ | P | $\chi^2$ | P |
| Axillary node negative | 62 | | NS | | NS |
| Axillary node positive | 63 | 6.77 | 0.0093 | 6.51 | 0.0107 |
| ER negative[a] | 56 | 7.28 | 0.0070 | 9.26 | 0.0026 |
| ER positive[a] | 86 | | NS | | NS |
| Tumour size ≤ 2 cm | 39 | | NS | | NS |
| Tumour size > 2 cm | 86 | | NS | | NS |
| uPA low | 107 | | NS | | NS |
| uPA high | 41 | 7.42 | 0.0064 | 9.67 | 0.0019 |

The cut-off point used for PAI-2 was 0.1 ng mg$^{-1}$ protein. Results are based on univariate analysis and the log-rank test. [a]The differences in the number of patients who are ER positive and negative in this table, compared with Table 1, are due to 2 patients on whom follow-up data were unavailable.

Table 4 compares the prognostic value of PAI-2 with uPA, PAI-1 and tPA. Using univariate analysis, the prognostic impact of PAI-2 was less strong than the other three parameters. However, in multivariate analysis, PAI-2 as a prognostic marker was independent of uPA, PAI-1 and tPA. Similarly, uPA, PAI-1 and tPA were independent prognostic factors. uPAR was not included in this comparative study, as results for this component were only available on 115 primary cancers. Finally, PAI-2 was investigated for possible prognostic value in different subgroups of patients with breast cancer (Table 5). Using the optimum cut-off point, PAI-2 levels were significantly related to outcome in the following subgroups: node-positive, ER-negative and patients with high levels of uPA (i.e. > 0.81 ng mg$^{-1}$ protein, which was the optimum cut-off point for discriminating between patients with good and poor outcome).

### DISCUSSION

Although PAI-1 has been widely studied in different human cancers, much less information is available on PAI-2. As with PAI-1 (Reilly et al, 1992), we show here that PAI-2 protein levels are also significantly correlated with those for uPA in breast cancer. However, the relationship between PAI-2 and uPA is less strong than that between PAI-1 and uPA. PAI-2 concentrations correlated weakly but significantly with those for PAI-1 levels at the protein but not at the mRNA level. Similar correlations between levels of PAI-2 protein and both uPA and PAI-1 have been reported by others (Foucre et al, 1991; Foekens et al, 1995). The lack of correlation at the mRNA level may be due to the smaller number of samples used for Northern blotting than for ELISA.

Using immunocytochemistry, we show that PAI-2 protein is found principally in malignant cells but is also present in stromal cells. Relatively little work appears to have been published on the detection of PAI-2 by immunocytochemistry in breast cancer. Using unprocessed tissue we previously found, using both monoclonal and polyclonal antibodies, that PAI-1 was mostly confined to tumour cells in breast cancer (Reilly et al, 1992). However, using paraffin-embedded tissue Bianchi et al (1995) found that PAI-1 was predominantly located in stromal cells, although epithelial cell staining was also present. In colon cancer, PAI-1 mRNA was found principally in endothelial cells. In this malignancy, no mRNA for PAI-1 was detected in the cancer cells (Pyke et al, 1991).

**Figure 5** **A** Relationship between PAI-2 levels and disease free interval using an optimum cut-off point of 0.1 ng mg$^{-1}$ protein. Differences in outcome were determined using the log-rank test, and the optimum cut-off point was calculated using the maximal log-rank test. There were eight (40%) relapses in the low PAI-2 group compared with 35 (27.34%) in the high PAI-2 group. Chi-square = 4.25; $P$ = 0.0392; RR = 1.91. **B** Relationship between PAI-2 levels and overall survival using an optimum cut-off point of 0.1 ng mg$^{-1}$ protein. Differences in outcome were determined using the log-rank test, and the optimum cut-off point was calculated using the maximal log-rank test. Chi-square = 6.45; $P$ = 0.0211; RR = 2.19

Our median levels for PAI-2 protein are lower than those previously reported (Foucre et al, 1991; Sumiyoshi et al, 1992; Foekens et al, 1995). These differences are probably related to the different assays used, i.e. different standards and different antibodies. However, our median values for PAI-2, PAI-1, uPA and uPAR are very similar to those previously reported by us (Duggan et al, 1995). In agreement with other investigators, we found no significant relationship between PAI-2 protein levels and established prognostic markers for breast cancer such as tumour size, nodal status and ER status (Foucre et al, 1991). Using the median value as a cut-off point, Foekens et al (1995) found no significant relationship between PAI-2 levels and either lymph node status or ER status, but did find that PAI-2 levels were significantly higher in smaller than larger tumours. In contrast to these findings, Sumiyoshi et al (1992) reported that PAI-2 levels were higher in carcinomas without than with nodal metastases.

 *© Cancer Research Campaign 1997*

The pathophysiological significance of the presence of both PAI-1 and PAI-2 in cancer is unclear. As one of the functions of these molecules is to inhibit uPA, it might be expected that high levels of these inhibitors would minimize cancer invasion and metastasis. In model systems, both PAI-1 and PAI-2 have indeed been shown to do this (see Introduction). However, in human breast cancer, high levels of PAI-1 are associated with poor prognosis (Grøndahl-Hansen et al, 1993; Jänicke et al, 1993; Foekens et al, 1994). These findings suggest that PAI-1 plays a role in the spread of cancer, perhaps by protecting the tumour from proteolysis during metastasis (Reilly et al, 1992).

Unlike PAI-1, high levels of PAI-2 do not appear to be associated with aggressive disease in breast cancer. Using an optimum cut-off point (i.e. 14.5 ng $mg^{-1}$ protein) Bouchet et al (1994) reported that high levels of PAI-2 were associated with both a longer disease-free interval and metastasis-free survival. In this study, however, only 14% of patients had high levels of PAI-2. In our study using an optimum cut-off of 0.1 ng $mg^{-1}$ protein, only 12.5% of patients had a low level of PAI-2. Foekens et al (1995) found no relationship between PAI-2 levels and patient outcome in the overall population. However, in patients with high levels of uPA, increasing PAI-2 levels were associated with improved prognosis. In this investigation we also show that PAI-2 levels correlate with outcome in patients with high uPA levels. However, we also show that PAI-2 is prognostic in other subgroups of patients generally associated with aggressive disease, i.e. node-positive and ER-negative patients. Our results with uPA, PAI-1 and tPA, reported here, are in agreement with published data. Many different groups have shown that both uPA (for review see Duffy, 1996) and PAI-1 (see above) are strong and independent prognostic factors in breast cancer. Using an immunoradiometric assay (IRMA), we have reported previously that high levels of tPA predicted improved outcome in patients with breast cancer (Duffy et al, 1988). It should be pointed out, however, that the optimum cut-off point found for tPA using the present ELISA is lower than that previously obtained using IRMA. Again, the differences will probably relate to different standards and antibodies used in the ELISA and IRMA. The optimum cut-off point found for uPA in the present study was identical to that used in the previous study using the American Diagnostica ELISA (Duggan et al, 1995).

We conclude that high PAI-2 levels, unlike those for PAI-1, are not associated with aggressive disease. On the other hand, high levels of PAI-2 may correlate with good prognosis, at least in certain subgroups of patients. These findings suggest that PAI-2 may be a favourable prognostic marker in breast cancer.

### ACKNOWLEDGEMENTS

This work was supported by the Irish Cancer Society, the International Association for Cancer Research, the Health Research Board of Ireland and the BIOMED 1 Programme of the European Union. (Project: Clinical Relevance of Proteases in Tumor Invasion and Metastasis, contract no. CT931346).

### REFERENCES

Andreasen P, Georg B, Lund LR, Riccio R and Stacey S (1990) Plasminogen activator inhibitors: hormonally regulated serpins. *Mol Cell Endocrinol* **68**: 1–19

Åstedt B, Lecander I and Ny T (1987) Placental type plasminogen activator inhibitor, PAI-2. *Fibrinolysis* **1**: 203–208

Åstedt B, Billström A, Lecander I (1995) Urokinase-producing tumour growth in SCID mice inhibited by recombinant PAI-2. *Fibrinol* **9**: 175–177

Baker MS, Bleakley P, Woodrow GC, Doe WF (1990) Inhibition of cancer cell urokinase plasminogen activator by its specific inhibitor PAI-2 and subsequent effects on extracellular matrix degradation. *Cancer Res* **50**: 4676–4684

Bianchi E, Cohen RL, Dai A, Thor AT, Schuman MA, Smith HS (1995) Immunohistochemical localization of the plasminogen activator inhibitor-1 in breast cancer. *Int J Cancer* **60**: 597–603

Bouchet C, Spyratos F, Martin PM, Hacene K, Gentile A, Oglobine J (1994) Prognostic value of urokinase-type plasminogen activator (uPA) and plasminogen activator inhibitors PAI-1 and PAI-2 in breast carcinomas. *Br J Cancer* **69**: 398–405

Cajot JF, Bamat J, Bergonzelli GE, Kruithof E, Medcalf RL, Testuz J, Gordat B (1990) Plasminogen-activator inhibitor type-1 is a potent natural inhibitor of extracellular matrix degradation by fibrosarcoma and colon carcinoma cells. *Proc Natl Acad Sci USA* **87**: 6939–6943

Chomczynski P, Sacchi N (1987) Single step method of RNA isolation by acid guanidinium thiocyanate-phenol-chloroform extraction. *Anal Biochem* **162**: 156–159

Duffy MJ (1993) Urokinase-type plasminogen activator and malignancy. *Fibrinolysis* **7**: 295–302

Duffy MJ (1996) Proteases as prognostic markers in cancer. *J Clin Cancer Res* **2**: 613–618

Duffy MJ, O'Siorain L, Waldron B, Smith C (1986) Estradiol receptor in human breast carcinomas assayed by use of monoclonal antibodies. *Clin Chem* **32**: 1972–1974

Duffy MJ, O'Grady P Devaney D, O'Siorain L, Fennelly JJ, Lijnen HR (1988) Tissue-type plasminogen activator: a new prognostic marker in breast cancer. *Cancer Res* **48**: 1348–1349

Duggan C, Maguire T, McDermott E, O'Higgins N, Fennelly JJ, Duffy MJ (1995) Urokinase plasminogen activator and urokinase plasminogen activator receptor in breast cancer. *Int J Cancer* **61**: 597–600

Foekens JA, Schmitt M, van Putten WLJ, Peters HA, Kramer M, Jänicke F, Klijn JGM (1994) Plasminogen activator inhibitor-1 and prognosis in primary breast cancer. *J Clin Oncol* **12**: 1648–1658

Foekens JA, Buessekler F, Peters HA, Krainick U, van Putten W, Look MP, Klijn JGM, Kramer M (1995) Plasminogen activator inhibitor 2: prognostic relevance in 1012 patients with primary breast cancer. *Cancer Res* **55**: 1423–1427

Foucre D, Bouchet C, Hacene K, Pourreau-Schneider N, Gentile A, Martin PM, Desplaces A, Oglobine J (1991) Relationship between Cathepsin D, urokinase and plasminogen activator inhibitors in malignant vs benign breast tumours. *Br J Cancer* **64**: 926–932

Grøndahl-Hansen J, Christensen IJ, Rosenquist C, Brünner N, Mouridsen HT, Danø K, Blichert-Toft M (1993) High levels of urokinase-type plasminogen activator and its inhibitor PAI-1 in cytosolic extracts of breast carcinomas are associated with poor prognosis. *Cancer Res* **53**: 2513–2521

Jänicke F, Schmitt M, Pache L, Ulm K, Harbeck N, Hofler H, Graeff H (1993) Urokinase (uPA) and its inhibitor PAI-1 are strong and independent prognostic factors in node negative breast cancer. *Breast Cancer Res Treat* **24**: 195–208

Mueller B, Yu YB, Laug W (1995) Overexpression of plasminogen activator inhibitor 2 in human melanoma cells inhibits spontaneous metastasis in *scid/scid* mice. *Proc Natl Acad Sci USA* **92**: 205–209

Pyke C, Kristensen P, Ralfkiaer E, Eriksen J, Danø K (1991) The plasminogen activator system in human colon cancer: messenger RNA for the inhibitor PAI-1 is located in endothelial cells in the human stroma. *Cancer Res* **51**: 4067–4071

Reilly D, Christensen L, Duch M, Nolan N, Duffy MJ, Andreasen P (1992) Type 1 plasminogen activator inhibitor in human breast carcinomas. *Int J Cancer* **50**: 208–214

Soff GA, Sanderowitz J, Gately S, Verrusio E, Weiss I, Brem S, Kwann HC (1995) Expression of plasminogen activator inhibitor type 1 by human prostate carcinoma cells inhibits primary tumor growth, tumor associated angiogenesis, and metastasis to lung and liver in an athymic mouse model. *J Clin Invest* **96**: 2593–2600

Sumiyoshi K, Serizawa K, Urano T, Takada Y, Takada A and Baba S (1992) Plasminogen activator system in human breast cancer. *Int J Cancer* **50**: 345–348

© Cancer Research Campaign 1997